# Lower Body Functional Test Performance Varies Based on Patient Demographics

**DOI:** 10.7759/cureus.116489

**Published:** 2026-09-18

**Authors:** Devin K Kelly, Amelia S Bruce Leicht, Xavier D Thompson, Gabrielle M DelBiondo, Jordan Hodges, Joe Hart

**Affiliations:** 1 Orthopedics, University of North Carolina at Chapel Hill, Chapel Hill, USA; 2 Athletic Training and Clinical Nutrition, University of Kentucky, Lexington, USA; 3 Orthopedics, School of Kinesiology, Louisiana State University, Baton Rouge, USA; 4 Orthopedic Surgery, Albany Medical College, Albany, USA

**Keywords:** hop testing, isokinetic knee extension, isometric knee extension, physical performance, return to sport

## Abstract

Background and aim: The utility of precise and instrumented musculoskeletal performance tests is essential to characterize musculoskeletal function during physical activity. Variation in these tests based on common patient demographics warrants consideration when using test results to counsel and advise physically active people. This study aimed to compare isolated muscle strength and functional performance outcomes by sex (male, female), age (late-stage adolescents: 18-21 years; adults: 22-34 years), and activity level (Tegner Activity Scale {TAS}: TAS ≥7, TAS <7). We hypothesized that males, adults, and individuals with higher activity levels would outperform their dichotomous pair.

Methods: This is a cross-sectional study. A total of 185 healthy participants (sex: 112 female, 73 male; age: 21±3 years; height: 173±10 cm; mass: 71±12 kg) with no history of lower extremity surgery and no history of lower extremity injury within the previous six months participated in this study. Participants completed isokinetic knee extension/flexion at 90°/s and 180°/s, isometric knee extension/flexion at 90° of knee flexion, the single-leg hop for distance, the triple hop for distance, and the 6-m timed hop.

Results: Males, adults, and participants with higher activity levels were stronger than females, late-stage adolescents, and participants with lower activity levels across all isolated muscle strength tests (p<0.05). Males, adults, and participants with higher activity levels jumped farther and faster than females, late-stage adolescents, and participants with lower activity levels for all hop tests except the triple hop for distance.

Conclusion: Males, adults, and participants with higher activity levels consistently outperformed females, late-stage adolescents, and participants with lower activity levels, highlighting differences in performance by sex, age, and activity level. These findings suggest that personalization should be considered when using musculoskeletal performance tests to make important healthcare decisions during recovery from injury and/or surgery.

## Introduction

Musculoskeletal performance assessments characterize strength and functional capabilities in athletes and patients. They may be used for the goals of maximizing sports performance or optimizing recovery from injury or surgery [1-6]. Precise and objective data from evidence-based assessment batteries are critical to identify treatable deficits and establish targets for physical training programs [1]. Previous research has demonstrated the utility of objective assessments to provide insight into recovery and potential injury risk, and aid the determination of readiness to return to unrestricted physical activity (RTA) [1-6].

Among many applications for evidence-based musculoskeletal assessment batteries, their use benefits patients recovering from anterior cruciate ligament reconstruction (ACLR) [5,7,8]. The objective data from these assessments support important clinical decisions regarding RTA, the timing of which is critical to mitigate reinjury risk [4,9-14]. However, current methods are ineffective as they may not relate to secondary injury risk [9]. Recovery after ACLR is typically based on side-to-side differences in strength and performance using the limb symmetry index (LSI) [2,10-14]. A “passing” LSI score of ≥90% is used as an indicator of success, but this can be misleading without considering the magnitude of performance metrics [2,10-14]. Moreover, current RTA criteria do not include intrinsic characteristics related to increased incidence of second contralateral ACL injury such as female sex, young age, family history of ACL injury, early reconstruction, and high activity level [15]. As such, the interpretation of performance results may be enhanced by accounting for the magnitude of metrics and personal characteristics of athletes and patients.

The purpose of this study was to compare muscle strength and functional performance outcomes in a cohort of uninjured participants based on sex, age, and activity level. We hypothesize that males, younger individuals, and individuals with higher activity levels will outperform their dichotomous pairs. Sex-, age-, and activity level-derived lower extremity performance data may inform targets for physically active people who want to enhance performance or improve function and provide a more accurate assessment of strength and functional capabilities.

## Materials and methods

Study design

This study was part of a large-scale point-of-care research project in patients with anterior cruciate ligament reconstruction (ACLR) and healthy controls at a single academic institution that evaluated strength, physical performance, and patient-reported outcomes as previously described [6,16-18]. Participants were stratified into dichotomous groups by sex, age, and activity level. Isokinetic peak knee extension and flexion torque at 90°/s and 180°/s and single-leg hop for distance, triple hop for distance, and 6-m timed hop were compared between groups. Age, sex, and activity level-specific performance data are reported.

Participants

The participants in the current study were included in the overall research project as healthy controls completing the same study protocol as ACLR patients. Participants were excluded if they had a history of lower extremity surgery or a history of lower extremity injury within the previous six months. A total of 185 healthy participants (sex: 112 female, 73 male; age: 21±3 years; height: 173±10 cm; mass: 71±12 kg) visited a research laboratory for a single testing session.

Procedures

After providing IRB-approved informed consent, each participant provided demographic information via a participant intake form. Next, the Tegner Activity Scale (TAS), which is free to use, was administered to determine participants' highest current activity levels [17]. It ranges from 0-10, with 0 indicating disability, 1-3 indicating low-demand activities of daily living, 4-6 indicating moderate activity levels such as recreational sports, and 7-10 indicating high-level competitive sports. Then participants warmed up by walking on a treadmill for 5 min at a standardized speed of 3 miles/h before completing the following performance tests: isokinetic knee extensor and flexor strength at 90°/s and 180°/s bilaterally, isometric extensor and flexor strength at 90° of knee flexion bilaterally, single-leg hop for distance, triple hop for distance, and 6-m timed hop [6,16-18].

Strength testing

Isolated knee extensor strength was measured during isokinetic and isometric strength testing using a multimode dynamometer (System 4; Shirley, NY: Biodex Medical Systems Inc.). All isometric data were collected using a universal data export to a data acquisition system (MP150; Goleta, CA: BIOPAC Systems Inc.) sampled at 125 Hz and low-pass filtered at 15 Hz for data processing as previously described [18]. For strength testing, participants were positioned in the dynamometer chair with their knees and hips flexed to 90°/s and 85°, respectively. For isokinetic trials, participants were instructed to cross their arms over their chest and extend and flex their knee with maximal effort for eight trials of concentric knee extension and flexion at both 90°/s and 180°/s, starting with their dominant limb followed by their nondominant limb. Participants were given visual feedback and verbal encouragement during isokinetic strength testing. The repetition that produced the highest peak torque (Nm) during extension and flexion was recorded as the peak knee extensor or flexor torque for each limb. For isometric testing, participants were instructed to cross their arms over their chest and extend or flex their knee with maximal effort for 30 s while positioned at 90° of knee flexion for their dominant limb, followed by their nondominant limb. Peak torque was calculated by obtaining the maximum 1 s average from the first 5 s of the test [7]. Participants were given familiarization trials prior to the start of data collection.

Functional testing

Functional tests were performed by completing three trials of each test per limb, starting with the dominant limb, and alternating between limbs for each trial. For the single-leg hop, participants were instructed to stand on one foot and hop as far forward as possible, landing on the same foot. For the triple hop, participants were instructed to quickly hop three times on one foot with the goal of achieving the greatest distance from the start line. Hop distance was measured using a 6-m measuring tape track as the distance from the start line to the back of the participant's heel upon landing. Hops for distance were considered successful if participants maintained their balance during the landing for 2 s. For the 6-m timed hop, participants were instructed to hop on one foot as fast as possible down a premeasured track for 6 m, with their times recorded for each trial using FITLIGHT (Aurora, ON: FITLIGHT Corp.). Participants were instructed to take as much rest as they needed between trials to avoid fatigue. The average of three trials per limb was calculated to determine hop distance for the single-leg and triple hops and hop time for the 6-m timed hop.

Statistical analysis

This study included a sample of convenience from a larger-scale study characterizing lower body muscle function in patients with knee injuries and healthy controls. Participants were stratified by sex (male; female), age (18-21 years; 22-34 years), and activity level (TAS <7; TAS ≥7). The age cutoff was determined according to the American Academy of Pediatrics definition of late-stage adolescence as a period of transition to adulthood occurring between ages 18 and 21 years. The activity-level cutoff was determined according to TAS >7, indicating competitive sports participation. Independent-samples t-tests were used to determine statistically significant differences between dichotomous groups for each characteristic (p<0.05). We then created participant categories for each combination of characteristics (e.g., males, age 18-21 years, TAS<7). Means, standard deviations, and 95% confidence intervals were calculated for all isolated muscle strength and functional performance tests and limb symmetry index (LSI) for each category. Cohen's d was calculated to determine the magnitude of differences, with values of 0.2, 0.5, and 0.8 interpreted as small, medium, and large effect sizes, respectively.

## Results

Males were stronger than females, adults were stronger than late-stage adolescents, and participants with higher activity levels were stronger than those with lower activity levels across nearly all isolated muscle strength tests (p<0.05) (Tables 1, 2) [17]. The exceptions to this were that individuals with higher activity levels were not significantly stronger than those with lower activity levels for peak knee extension at 90°/s or 180°/s. Sex, age, and activity level influenced functional performance outcomes (Table 3) [17]. Males hopped farther and faster than females across tests (p<0.05). Adults performed better on all hop tests compared to late-stage adolescents, but only the single hop was statistically significant (p<0.05). Participants with higher activity levels performed better on single and triple hop tests compared to participants with lower activity levels (p<0.05). Highlighted group means are presented in Figure 1.

**Table 1:** Isokinetic peak torques normalized to body mass (Nm/kg) by sex, age, and activity level (TAS). *Significant differences between groups according to independent-samples t-tests (p<0.05). d: Cohen's d effect size; TAS: Tegner Activity Scale

Variables		Isokinetic peak torque (Nm/kg)																
		n	Extension 90°/s, mean±SD (90% CI)	d	t	p-Value	Flexion 90°/s, mean±SD (90% CI)	d	t	p-Value	Extension 180°/s, mean±SD (90% CI)	d	t	p-Value	Flexion 180°/s, mean±SD (90% CI)	d	t	p-Value
Sex	Males	73	2.5±0.4* (2.4-2.6)	1.51	10.0	<0.001	1.1±0.3* (1.0-1.1)	0.75	4.99	<0.001	2.0±0.3* (1.9-2.0)	1.42	9.41	<0.001	0.8±0.2* (0.8-0.9)	0.64	4.27	<0.001
	Females	112	1.9±0.4* (1.9-2.0)				0.9±0.2* (0.9-0.9)				1.5±0.3* (1.5-1.6)				0.7±0.2* (0.7-0.7)			
Age (years)	18-21	133	2.1±0.4* (2.0-2.1)	0.75	4.59	<0.001	0.9±0.2* (0.9-1.0)	0.65	3.97	<0.001	1.6±0.4* (1.6-1.7)	0.63	3.85	<0.001	0.7±0.2* (0.7-0.8)	0.67	4.07	<0.001
	≥22	52	2.4±0.4* (2.3-2.5)				1.1±0.2* (1.0-1.1)				1.9±0.3* (1.8-1.9)				0.9±0.2* (0.8-0.9)			
TAS	<7	46	2.1±0.5 (1.9-2.2)	0.30	1.77	0.08	0.9±0.3* (0.8-1.0)	0.42	2.45	0.02	1.6±0.4 (1.5-1.7)	0.27	1.60	0.11	0.7±0.2* (0.6-0.7)	0.46	2.71	0.01
	≥7	139	2.2±0.5 (2.1-2.3)				1.0±0.2* (1.0-1.0)				1.7±0.4 (1.7-1.8)				0.8±0.2* (0.8-0.8)			

**Table 2:** Isometric peak torques normalized to body mass (Nm/kg) by sex, age, and activity level (TAS). *Significant differences between groups according to independent-samples t-tests (p<0.05). d: Cohen's d effect size; TAS: Tegner Activity Scale

Variables		Isometric peak torque (Nm/kg)								
		n	Extension 90°, mean±SD (90% CI)	d	t	p-Value	Flexion 90°, mean±SD (90% CI)	d	t	p-Value
Sex	Males	73	2.5±0.7* (2.3-2.6)	1.0	6.67	<0.001	1.0±0.3* (0.9-1.0)	0.68	4.55	<0.001
	Females	112	1.9±0.5* (1.8-2.0)				0.8±0.2* (0.8-0.8)			
Age (years)	18-21	133	2.0±0.6* (1.9-2.1)	0.49	2.97	<0.01	0.8±0.3* (0.8-0.9)	0.43	2.63	0.01
	≥22	52	2.3±0.7* (2.2-2.5)				1.0±0.2* (0.9-1.0)			
TAS	<7	46	1.9±0.6* (1.8-2.1)	0.38	2.21	0.03	0.8±0.3* (0.7-0.9)	0.38	2.26	0.03
	≥7	139	2.2±0.7* (2.1-2.3)				0.9±0.3* (0.9-0.9)			

**Table 3:** Hop testing distance and time normalized to height (m/m, s/m) by sex, age, and activity level (TAS). *Significant differences between groups according to independent-samples t-tests (p<0.05). d: Cohen's d effect size; TAS: Tegner Activity Scale

Variables		Hop testing - normalized by height												
		n	Single hop, mean±SD (90% CI)	d	t	p-Value	Triple hop, mean±SD (90% CI)	d	t	p-Value	6-m timed hop, mean±SD (90% CI)	d	t	p-Value
Sex	Males	73	0.86±0.13* (0.84-0.89)	1.41	9.35	<0.001	2.91±0.49* (2.81-3.0)	1.34	8.86	<0.001	0.01±0.0* (0.01 -0.01)	0.16	1.06	0.29
	Females	111	0.67±0.14* (0.65-0.69)				2.28±0.45* (2.21-2.35)				0.03±0.13* (0.01-0.05)			
Age (years)	18-21	132	0.73±0.16* (0.70-0.75)	0.41	2.50	0.01	2.49±0.55 (2.41-2.57)	0.25	1.55	0.12	0.02±0.12 (0.01-0.04)	0.12	0.71	0.48
	≥22	52	0.79±0.16* (0.76-0.83)				2.63±0.58 (2.49-2.77)				0.01±0.0 (0.01-0.01)			
TAS	<7	46	0.67±0.16* (0.63-0.70)	0.67	3.94	<0.001	2.29±0.56* (2.16-2.43)	0.58	3.39	<0.001	0.01±0.0 (0.01-0.02)	0.08	0.45	0.66
	≥7	138	0.77±0.16* (0.75-0.79)				2.61±0.54* (2.53-2.68)				0.02±0.12 (0.01-0.04)			

**Figure 1:**
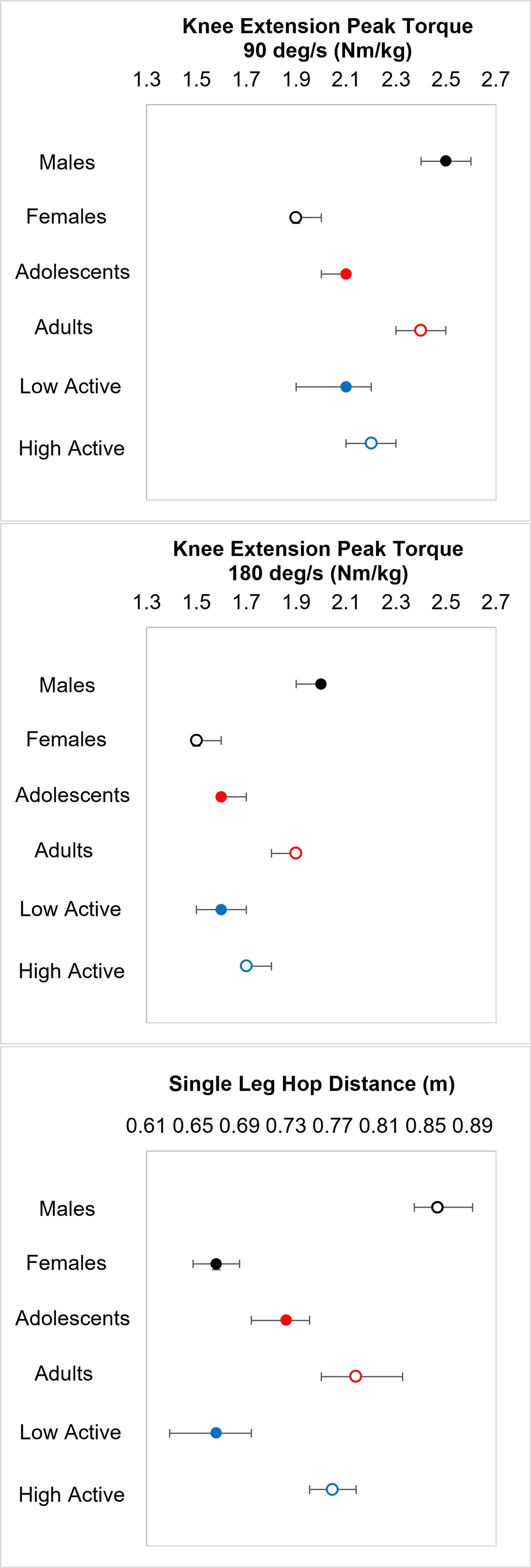
Strength and hop performance differences by sex, age, and activity level. Sex, age, and activity level group means and confidence intervals for knee extension peak torque at 90°/s normalized to body mass (Nm/kg) (top), knee extension peak torque at 180°/s normalized to body mass (Nm/kg) (middle), and single-leg hop distance (m) (bottom).

While individual characteristics influenced isolated muscle strength and functional performance outcomes, they generally did not influence LSI values (Tables 4-6) [17]. Females had more symmetric isokinetic peak flexor torque at 180°/s compared to males; however, males had more symmetric isometric peak flexor torque (p<0.05). Sex did not influence symmetry on any other test. Neither age nor activity level affected symmetry on any test.

**Table 4:** Limb symmetry indices for isokinetic peak torques normalized to body mass (Nm/kg) by sex, age, and activity level (TAS). *Significant differences between groups according to independent-samples t-tests (p<0.05). d: Cohen's d effect size; TAS: Tegner Activity Scale

Variables		Isokinetic peak torque limb symmetry index (%)																
		n	Extension 90°/s, mean±SD (90% CI)	d	t	p-Value	Flexion 90°/s, mean±SD (90% CI)	d	t	p-Value	Extension 180°/s, mean±SD (90% CI)	d	t	p-Value	Flexion 180°/s, mean±SD (90% CI)	d	t	p-Value
Sex	Males	73	99.9±11.5 (97.7-102.2)	0.14	0.92	0.36	95.8±12.8 (93.3-98.3)	0.24	1.61	0.11	98.9±11.5 (96.7-101.1)	0.03	0.18	0.86	96.1±16.0* (93.0-99.2)	0.33	2.22	0.03
	Females	112	98.5±9.9 (96.9-100.0)				99.3±15.5 (96.8-101.8)				99.2±12.1 (97.3-101.1)				101.0±15.5* (98.9-103.7)			
Age (years)	18-21	133	98.4±10.0 (96.9-99.8)	0.23	1.42	0.16	97.9±14.1 (95.9-99.9)	0.01	0.08	0.93	99.2±12.1 (97.4-100.9)	0.02	0.14	0.89	99.3±16.7 (96.9-101.7)	0.01	0.04	0.97
	≥22	52	100.8±11.9 (98.0-103.6)				98.1±15.9 (94.3-101.8)				98.9±11.1 (96.3-101.5)				99.2±13.7 (96.0-102.4)			
TAS	<7	46	97.9±10.3 (95.3-100.4)	0.14	0.85	0.40	98.9±18.7 (94.3-103.6)	0.10	0.55	0.59	99.3±10.0 (96.8-101.8)	0.02	0.13	0.90	98.9±16.4 (94.8-102.9)	0.03	0.19	0.85
	≥7	139	99.4±10.7 (97.9-100.9)				97.6±13.0 (95.8-99.4)				99.0±12.4 (97.3-100.80				99.4±15.7 (97.2-101.6)			

**Table 5:** Limb symmetry indices for isometric peak torques normalized to body mass (Nm/kg) by sex, age, activity level (TAS). *Significant differences between groups according to independent-samples t-tests (p<0.05). d: Cohen's d effect size; TAS: Tegner Activity Scale

Variables		Isometric peak torque limb symmetry index (%)								
		n	Extension 90°, mean±SD (90% CI)	d	t	p-Value	Flexion 90°, mean±SD (90% CI)	d	t	p-Value
Sex	Males	73	98.0±26.0 (92.9-103.0)	0.25	1.65	0.10	100.4±26.0* (95.4-105.5)	0.32	2.11	0.04
	Females	112	92.5±18.9 (89.5-95.5)				109.0±25.7* (104.6-112.7)			
Age (years)	18-21	133	93.6±21.2 (90.6-96.7)	0.16	1.01	0.32	106.0±27.2 (102.1-110.0)	0.09	0.54	0.59
	≥22	52	97.3±24.1 (91.7-102.9)				103.7±23.0 (98.4-109.1)			
TAS	<7	46	92.0±21.4 (86.7-97.3)	0.16	0.94	0.35	104.5±22.8 (98.8-110.1)	0.05	0.28	0.78
	≥7	139	95.5±22.3 (92.4-98.7)				105.7±27.1 (100.9-109.5)			

**Table 6:** Limb symmetry indices for hop testing distance and time normalized to height (m/m; s/m) by sex, age, activity level (TAS). d: Cohen's d effect size; TAS: Tegner Activity Scale

Variables		Limb symmetry index - hop testing (%)												
		n	Single hop, mean±SD (90% CI)	d	t	p-Value	Triple hop, mean±SD (90% CI)	d	t	p-Value	6-m timed hop, mean±SD (90% CI)	d	t	p-Value
Sex	Males	73	100.3±5.9 (99.1-101.4)	0.03	0.23	0.82	99.2±8.2 (97.6-100.8)	0.07	0.47	0.64	100.5±9.2 (98.7-102.3)	0.13	0.88	0.38
	Females	111	100.0±7.2 (98.9-101.2)				98.6±8.3 (97.2-99.9)				99.5±6.7 (98.4-100.5)			
Age (years)	18-21	132	100.0±6.9 (98.9-100.9)	0.10	0.61	0.54	98.9±8.2 (97.7-100.0)	0.02	0.14	0.89	100.2±8.3 (99.0-101.4)	0.13	0.77	0.45
	≥22	52	100.6±6.1 (99.2-102.0)				98.7±8.7 (96.7-100.7)				99.2±6.5 (97.7-100.7)			
TAS	<7	46	100.7±8.3 (98.7-102.8)	0.12	0.68	0.50	98.9±5.8 (97.4-100.3)	0.01	0.08	0.94	99.1±5.2 (97.8-100.4)	0.14	0.80	0.42
	≥7	138	99.9±6.1 (99.1-100.8)				98.8±9.0 (97.5-100.0)				100.2±8.5 (99.0-100.8)			

Given these findings, appendix presents characteristic-specific performance outcomes for isolated muscle strength and functional tests. Characteristic-specific performance values for isokinetic and isometric knee extensor and flexor strength outcomes are presented as both absolute and normalized to body mass. Characteristic-specific hop-testing performance values are presented as both absolute and normalized to height. All characteristic-specific performance values presented are derived from the nondominant limb as a conservative reference, with the injured and contralateral limbs of a patient group corresponding to the nondominant and dominant limbs of a control group.

## Discussion

The primary findings of this study are that healthy individuals' sex, age, and activity level nonuniformly differentiate performance on lower-body musculoskeletal strength and performance tests. In general, we found that males, adults, and participants with higher activity levels outperformed females, late-stage adolescents, and participants with lower activity levels. Caution should be exercised when interpreting data from lower-body musculoskeletal tests as the basis for counseling patients or clients or making healthcare decisions. Mixed cohorts of healthy individuals in the literature may be inappropriate comparators because they may overestimate or underestimate an individual patient's or client's performance capacity relative to the cohort. The differences observed in performance and strength data support the need for demographics-based personalization of performance thresholds that will help people seeking functional gains through exercise interventions.

Since the tests used in the current study are commonly used in recovery from ACLR, there are applications of these findings for clinicians who are making data-based recommendations about rehabilitation progressions and timing of clearance to return to unrestricted physical activity (RTA). Results from the present study in healthy individuals exhibit similar between-sex and between-age differences in performance that have been observed in mixed cohorts of individuals recovering from ACLR. We found that males produced greater peak torque across isokinetic and isometric tests and performed better on most hop tests compared to females. Similarly, age differentiated outcomes on all tests in the present study, with adults performing better compared to late-stage adolescents. Previously, males have been shown to be stronger than females post-ACLR in early adolescence through adulthood and across time post-operatively [16,19-21]. When comparing strength post-ACLR, adults aged 18-40 years were stronger than adolescents aged 13-17 years [20]. Because individuals recovering from ACLR will focus rehabilitation on recovery of strength and functional performance based on healthy individuals, thresholds should be sex- and age-based. Thus, the results presented may provide improved performance targets.

Strength and performance measures of healthy individuals were not as clearly differentiated by activity level, based on the Tegner Activity Scale, as they were for sex and age. Our results showed that healthy individuals with higher activity levels were stronger only in flexion during isokinetic testing and in both extension and flexion for isometric testing compared to individuals with lower activity levels. Individuals with higher activity levels also performed better on single- and triple-hop testing, but not the 6-m timed hop compared to lower activity levels. Using the Tegner Activity Scale to determine activity-level-based performance thresholds may have produced a ceiling effect, as most participants reported a Tegner score of ≥7. Moreover, this scale or dichotomous grouping may not account for differences present in high activity levels, such as differing demands across levels and types of competitive sports. Evaluation of healthy individual performance showed that athletes participating in the National Collegiate Athletic Association (NCAA) Division I athletics compared to recreational or competitive athletes not participating in NCAA Division I athletics were stronger for some but not all strength measures and performed better on hop testing, suggesting that nuanced activity level differences should be considered in defining performance thresholds [21]. Interestingly, although hop test outcomes are strongly correlated, triple hop did not differ by age or activity level, which may indicate reduced test reliability [22]. Future work may determine normative thresholds that are competitive level and sport-specific.

Limb symmetry index values across strength and functional performance tests were relatively less dependent upon individual characteristics. This may be due to the variance in LSI values. For example, the standard deviations for LSI isometric peak extension at 90° across all dichotomous groups were greater than 20%. This finding suggests that there is inherent between-limb variability in a healthy cohort. Future work should investigate appropriate LSI thresholds according to each test that account for variance in healthy movement.

Limitations

We limited comparisons to age, sex, and activity level since those factors have impacted recovery and risk for future injury after primary ACLR. Other factors may influence values; however, because of our limited convenience sample, we compared only these variables. The order of tests was not randomized in the present study, which may impact results; however, they were implemented in the progression that individuals recovering from ACLR would be tested in the RTA assessment. Additionally, the statistical analysis did not apply Bonferroni correction for multiple comparisons, which may increase the risk of false positives. Alternatively, a conservative approach may hide true differences in a relatively small sample. For improved interpretation of the results, effect sizes and confidence intervals were reported. There may be other person-specific features that were not captured in this study that may also impact variability in strength and performance metrics measured in the current study. Future studies with larger sample sizes may consider other factors with potential relevance to patients recovering from ACLR or other musculoskeletal injuries.

Applications to practice

The results from the current study demonstrate the need for personalization in outcome metrics from musculoskeletal performance evaluations based on age, sex, and activity level. A patient recovering from a knee injury or surgery can use data from the current study to set strength and performance goals based on data from uninjured people with similar age, sex, and activity level. For example, a young, highly active female recovering from ACLR should consider values based on their specific sex, age, and activity level when making decisions about progressions through stages of post-operative recovery. While these metrics can provide a performance target based on healthy individuals and may inform recovery progress, they are not definitive post-ACLR RTA criteria.

## Conclusions

The findings from this study demonstrate the importance of considering the impact of individual characteristics, including sex, age, and activity level, on performance outcomes from common musculoskeletal performance assessments. In general, male sex and adult age resulted in better strength and functional performance across tests. Higher activity level resulted in better functional performance on hop testing. Using personalized values for performance may help inform training goal setting, interpretation of outcomes, and recovery progression following injury or surgery, which may inform clinical decision-making.

## Appendices

**Table 7:** Normative reference values for isokinetic peak torques normalized to body mass (Nm/kg) according to sex (male, female), age (18-21 years: late adolescence, ≥22 years: adulthood), and TAS (<7: low-to-moderately active, ≥7: highly active). SEM: standard error of measurement; TAS: Tegner Activity Scale

Variables				Isokinetic peak torque (Nm)											
				Extension 90°/s			Flexion 90°/s			Extension 180°/s			Flexion 180°/s		
Sex	Age (years)	TAS	n	Mean±SD	SEM	90% CI	Mean±SD	SEM	90% CI	Mean±SD	SEM	90% CI	Mean±SD	SEM	90% CI
Male	18-21	≥7	38	180.2±42.1	6.8	168.6-191.7	78.1±24.2	3.9	71.4-84.7	141.9±33.7	5.5	132.7-151.1	60.6±21.8	3.5	54.7-66.6
		<7	3	183.0±9.0	5.2	167.8-198.2	63.3±14.8	8.6	38.4-88.3	139.0±10.4	6.0	121.4-156.6	47.4±7.3	4.2	35.1-59.8
	≥22	≥7	22	210.3±43.8	9.3	194.3-226.4	91.6±20.3	4.3	84.1-99.0	165.1±34.1	7.3	152.6-177.6	73.8±17.0	3.6	67.5-80.0
		<7	10	197.2±43.0	13.6	172.3-222.1	86.7±26.4	8.4	71.3-102.0	153.4±35.9	11.4	132.6-174.3	67.3±19.0	6.0	56.3-78.3
Female	18-21	≥7	64	132.4±31.6	3.9	125.8-139.0	62.5±15.1	1.9	59.4-65.7	103.2±30.2	3.8	96.9-109.5	49.2±15.6	2.0	45.9-52.5
		<7	28	119.1±24.7	4.7	111.2-127.0	52.7±11.6	2.2	49.0-56.5	93.2±20.3	3.8	86.7-99.7	40.4±8.7	1.6	37.6-43.3
	≥22	≥7	15	131.3±25.3	6.5	119.8-142.8	64.1±12.6	3.3	58.4-69.9	98.7±24.5	6.3	87.5-109.8	51.4±12.5	3.2	45.8-57.1
		<7	5	133.5±34.8	15.6	100.3-166.7	60.2±15.0	6.7	45.9-74.5	104.9±17.8	8.0	87.9-121.9	47.2±8.6	3.9	38.9-55.4

**Table 8:** Characteristic-specific values for isometric knee extension and flexion at 90° by sex (male, female), age (18-21 years: late adolescence, ≥22 years: adulthood), and TAS (<7: low-to-moderately active, ≥7: highly active). SEM: standard error of measurement; TAS: Tegner Activity Scale

Variables				Isometric peak torque (Nm)					
				Extension 90°			Flexion 90°		
Sex	Age (years)	TAS	n	Mean±SD	SEM	90% CI	Mean±SD	SEM	90% CI
Male	18-21	≥7	38	179.9±57.0	9.2	164.3-195.5	73.3±25.0	4.0	66.5-80.2
		<7	3	179.3±49.4	28.5	96.0-262.6	62.1±26.3	15.2	17.7-106.5
	≥22	≥7	22	219.9±67.6	14.4	195.1-244.7	80.1±17.7	3.8	73.6-86.6
		<7	10	173.3±68.3	21.6	133.7-212.9	70.9±21.2	6.7	58.6-83.2
Female	18-21	≥7	64	128.5±37.7	4.7	120.7-136.4	54.6±14.5	1.8	51.6-57.6
		<7	28	114.9±32.8	6.2	104.3-125.5	46.7±11.2	2.1	43.1-50.3
	≥22	≥7	15	125.8±30.8	8.0	111.8-139.8	57.3±13.2	3.4	51.3-63.3
		<7	5	123.6±36.3	16.2	89.0-158.2	57.3±16.2	7.2	41.9-72.7

**Table 9:** Characteristic-specific values for isokinetic knee extension and flexion at 90°/s and 180°/s by sex (male, female), age (18-21 years: late adolescence, ≥22 years: adulthood), and TAS (<7: low-to-moderately active, ≥7: highly active). SEM: standard error of measurement; TAS: Tegner Activity Scale

Variables				Isokinetic peak torque (Nm/kg)											
				Extension 90°/s			Flexion 90°/s			Extension 180°/s			Flexion 180°/s		
Sex	Age (years)	TAS	n	Mean±SD	SEM	90% CI	Mean±SD	SEM	90% CI	Mean±SD	SEM	90% CI	Mean±SD	SEM	90% CI
Male	18-21	≥7	38	2.4±0.4	0.06	2.3-2.5	1.0±0.3	0.04	1.0-1.1	1.9±0.3	0.05	1.8-2.0	0.8±0.2	0.04	0.7-0.9
		<7	3	2.4±0.5	0.31	1.5-3.3	0.9±0.4	0.20	0.2-1.5	1.8±0.4	0.22	1.2-2.5	0.6±0.2	0.10	0.4-0.9
	≥22	≥7	22	2.6±0.4	0.08	2.4-2.7	1.1±0.2	0.05	1.0-1.2	2.0±0.3	0.07	1.9-2.1	0.9±0.2	0.04	0.8-1.0
		<7	10	2.6±0.4	0.12	2.4-2.8	1.1±0.3	0.09	1.0-1.3	2.0±0.3	0.09	1.8-2.2	0.9±0.2	0.06	0.8-1.0
Female	18-21	≥7	64	1.9±0.4	0.05	1.9-2.0	0.9±0.2	0.02	0.9-1.0	1.5±0.4	0.05	1.4-1.6	0.7±0.2	0.03	0.7-0.8
		<7	28	1.8±0.3	0.06	1.7-1.9	0.8±0.2	0.04	0.7-0.9	1.4±0.3	0.05	1.3-1.5	0.6±0.1	0.02	0.6-0.7
	≥22	≥7	15	2.1±0.2	0.05	2.0-2.2	1.0±0.1	0.04	0.9-1.1	1.5±0.2	0.06	1.4-1.6	0.8±0.2	0.04	0.7-0.9
		<7	5	2.2±0.4	0.18	1.8-2.5	1.0±0.1	0.06	0.8-1.1	1.7±0.1	0.06	1.6-1.8	0.8±0.1	0.03	0.7-0.8

**Table 10:** Normative reference values for isometric peak torques normalized to body mass (Nm/kg) according to sex (male, female), age (18-21 years: late adolescence, ≥22 years: adulthood), and TAS (<7: low-to-moderately active, ≥7: highly active). SEM: standard error of measurement; TAS: Tegner Activity Scale

Variables				Isometric peak torque (Nm/kg)					
				Extension 90°			Flexion 90°		
Sex	Age (years)	TAS	n	Mean±SD	SEM	90% CI	Mean±SD	SEM	90% CI
Male	18-21	≥7	38	2.4±0.7	0.11	2.2-2.6	1.0±0.3	0.05	0.9-1.1
		<7	3	2.4±0.9	0.54	0.8-4.0	0.9±0.6	0.34	0.0-1.9
	≥22	≥7	22	2.7±0.7	0.16	2.4-3.0	1.0±0.2	0.05	0.9-1.1
		<7	10	2.3±0.8	0.24	1.8-2.7	0.9±0.2	0.08	0.8-1.1
Female	18-21	≥7	64	1.9±0.5	0.07	1.8-2.0	0.8±0.2	0.03	0.8-0.9
		<7	28	1.7±0.5	0.10	1.6-1.9	0.7±0.2	0.04	0.7-0.8
	≥22	≥7	15	2.0±0.4	0.10	1.8-2.2	0.9±0.2	0.05	0.8-1.0
		<7	5	2.0±0.6	0.28	1.4-2.6	1.0±0.3	0.14	0.6-1.3

**Table 11:** Characteristic-specific values for single hop (m), triple hop (m), and 6-m timed hop (s) by sex (male, female), age (18-21 years: late adolescence, ≥22 years: adulthood), and TAS (<7: low-to-moderately active, ≥7: highly active). SEM: standard error of measurement; TAS: Tegner Activity Scale

Variables				Hop testing								
				Single hop			Triple hop			6-m timed hop		
Sex	Age (years)	TAS	n	Mean±SD	SEM	90% CI	Mean±SD	SEM	90% CI	Mean±SD	SEM	90% CI
Male	18-21	≥7	38	1.54±0.25	0.04	1.48-1.61	5.27±0.88	0.14	5.03-5.51	1.86±0.28	0.04	1.79-1.94
		<7	3	1.32±0.35	0.20	0.73-1.92	4.60±1.21	0.70	2.56-6.63	2.46±0.87	0.50	1.00-3.93
	≥22	≥7	22	1.61±0.26	0.05	1.51-1.70	5.17±1.07	0.23	4.77-5.56	1.96±0.28	0.06	1.85-2.06
		<7	10	1.50±0.18	0.06	1.40-1.61	5.33±0.58	0.18	5.00-5.67	1.96±0.31	0.10	1.78-2.14
Female	18-21	≥7	63	1.19±0.23	0.03	1.14-1.23	4.03±0.76	0.10	3.87-4.19	2.35±0.42	0.05	2.26-2.43
		<7	28	0.98±0.22	0.04	0.91-1.05	3.45±0.70	0.13	3.22-3.67	2.68±0.55	0.10	2.51-2.86
	≥22	≥7	15	1.12±0.17	0.05	1.04-1.20	3.90±0.55	0.14	3.65-4.15	2.37±0.24	0.06	2.26-2.49
		<7	5	1.13±0.17	0.08	0.97-1.29	3.25±0.94	0.42	2.36-4.15	2.31±0.24	0.11	2.09-2.54

**Table 12:** Normative reference values for hop distances and time normalized to height (m/m, s/m) according to sex (male, female), age (18-21 years: late adolescence, ≥22: years: adulthood), and TAS (<7: low-to-moderately active, ≥7: highly active). SEM: standard error of measurement; TAS: Tegner Activity Scale

Variables				Hop testing - normalized by height								
				Single hop			Triple hop			6-m timed hop		
Sex	Age (years)	TAS	n	Mean±SD	SEM	90% CI	Mean±SD	SEM	90% CI	Mean±SD	SEM	90% CI
Male	18-21	≥7	38	0.86±0.13	0.02	0.83-0.90	2.95±0.45	0.07	2.83-3.07	0.011±0.002	0	0.010-0.011
		<7	3	0.75±0.22	0.13	0.38-1.12	2.60±0.76	0.44	1.32-3.88	0.014±0.005	0.003	0.006-0.022
	≥22	≥7	22	0.88±0.15	0.03	0.83-0.94	2.84±0.61	0.13	2.61-3.06	0.011±0.001	0	0.010-0.011
		<7	10	0.84±0.10	0.03	0.78-0.89	2.97±0.29	0.09	2.80-3.14	0.011±0.002	0.001	0.010-0.012
Female	18-21	≥7	63	0.70±0.14	0.02	0.67-0.73	2.39±0.46	0.06	2.29-2.48	0.036±0.173	0.022	0.0-0.073
		<7	28	0.59±0.12	0.02	0.55-0.63	2.07±0.38	0.07	1.95-2.20	0.016±0.004	0.001	0.015-0.017
	≥22	≥7	15	0.67±0.13	0.03	0.61-0.73	2.32±0.37	0.10	2.15-2.49	0.014±0.002	0	0.013-0.015
		<7	5	0.69±0.10	0.04	0.59-0.78	1.98±0.55	0.25	1.45-2.50	0.014±0.001	0.001	0.013-0.016
